# Supplementary material for: Dogs and Their Owners Have Frequent and Intensive Contact
Source: Int J Environ Res Public Health. 2020 Jun 16;17(12):4300. doi: 10.3390/ijerph17124300 (PMC7345801; doi:10.3390/ijerph17124300)
Supplement: Supplementary file 1 [file ijerph-17-04300-s001.pdf]

Supplementary Materials Part A: Questionnaire

This questionnaire is a translation from the original questionnaire that was drafted in Dutch.

Contact between dog and its owner

Welcom

Dear participant,

This questionnaire consists out of 3 parts, where we will ask for information regarding your family, your dog and the contact between you and your dog. This survey is set up to make 1st bachelor students of Veterinary Medicine, who are following the Epidemiology course, more familiar with conducting surveys. The results of this survey will be analyzed anonymously. Thank you for finding the time to fill in this questionnaire.

Kind regards,

Contact between dog and its owner

General informlation of the owner

* 1. What is your gender?

- Male
- Female
- X

* 2. What is your age?*(in years)*

* 3. What is the composition of your family: How many adults live under the same roof? (including you, when applicable)

* 4. What is the composition of your family: How many children live under the same roof? (including you, when applicable)

* 5. I live

- In a city
- In a village
- in the countryside
- other

* 6. What is your highest level of education?

- Elementary school
- High school
- College
- University
- Other (please clarify)

* 7. Do you work?

- y e s , f u l l

time

◯ yes, part-time

no

8. Do you come into contact with animals through your profession?

◯ yes

◯ no

9. With what species do you come into contact through your profession? (e.g. veterinarian, farmer, dog trainer, …) *(multiple answers possible)*

☐ None

☐ Dogs

☐

☐

☐

☐

C
a
t
s
H
o
r
s
e
s
C
a
t
t
l
e
S
h
e
e
p
o
r
g
o
a
t
s
C
h
i

c
k
e
n
P
i
g
s
R
a
b
b
i
t
s
O
t
h
e
r
:

10. Do you have to visit hospitals to practice your profession?

yes, annually

y
e
s
,
m
o
n
t
h

ly

○ yes, weekly

○ yes, daily

○ No

* 11. Do you study?

- ◯ yes
- ◯ no

12. Do you come into contact with animals through your studies?

- ◯ yes
- ◯ no

13. With what species do you come into contact through your studies? (e.g. veterinarian, farmer, dog trainer, …) *(multiple answers possible)*

- ☐ None
- ☐ Dogs
- ☐ Cats
- ☐ Hor
- ☐
- ☐

s
e
s
C
a
t
t
l
e
S
☐ h
☐ e
e
p
o
r
g
o
a
t
s
C
h
i
c
k
e
n
P
☐ i
☐ g
s

R

a

b

b

i

t

s

O

t

h

e

r

:

14. Do you have a dog?

Y

e

s

N

o

15. What species do you have as a pet? Please also write down the number of animals.

Guinea Pig	
Duck	
Goat	
Dog	
Cat	
Chicken	
Rabbit	
Mouse	
Horse	
Rat	
Reptile	
Cattle	
Sheep	
Pig	
Fish	
Bird	
Other:	

16. Did you receive a treatment with antibiotics in the past 6 months?

○ y
e
s

○ N
o

17. Are you pregnant or do you have a medical condition resulting in a weakened immune system (diabetic, cancer, Leukemia)?

○

○ y

es

No

In the next part of the questionnaire, questions will be asked regarding your dog. If you have more than one dog, then please select one to fill in the questionnaire.

UNIVERSITEIT GENT

FACULTEIT DIERGENEESKUNDE
accredited by EAEVE

Contact between dog and its owner

General information of the dog

18. What is the gender of your dog?

- ○ Male dog
- ○ Bitch (female)

19. Is your dog castrated/spayed?

◯

y

e

s

◯

n

o

20. How old Is your dog? Enter the age in years, rounding up. (e.g. a pup of 6 months ➔ enter 1 year)

☐

21. How big is your dog?

◯ small

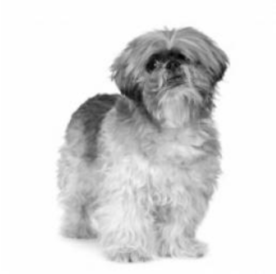

◯ Medium

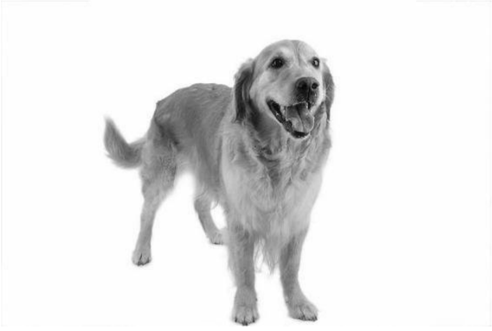

◯ Large

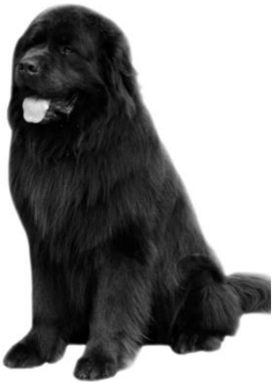

22. What is the main function of your dog? *(multiple answers possible)*

☐ C

☐ o

☐ m

☐

☐

☐

p
a
n
i
o
n
a
n
i
m
a
l
A
s
s
i
s
t
i
n
g
d
o
g
H
u
n
t
i
n
g
d

o
g
G
u
a
r
d
d
o
g
P
o
l
i
c
e
d
o
g
R
e
s
c
u
e
d
o
g
O
t
h
e

r

:

23. Does your dog gets vaccinated at least every three years?

- ◯ yes
- ◯ no

24. How many times a year does your dog gets dewormed? (if you never do this, then please enter 0)

25. How many times a year does your dog gets treated for fleas? (if you never do this, then please enter 0)

26. Where is your dog allowed when you are at home? *(multiple answers possible)*

- ☐ Everywhere in the house
- ☐ Certain parts of the house
- ☐ The whole garden
- ☐ Certain parts of the

garden

Kitchen

Living

room

Bedroom

Bench

☐ other

(please

specify):

27. Where is your dog allowed when you are NOT at home? *(multiple answers possible)*

☐ Everywh

☐ ere in the

☐ house

☐ Certain

☐ parts of

☐ the

☐ house

☐ The

whole

garden

Certain

parts of

the

garden

Kitchen

Living

room

Bedroom

Bench

☐ other

(please

specify):

28. What is your dog's main diet? *(multiple answers possible)*

☐ Commercial dry feed (e.g.

Royal Canin, Hills,…)

☐ Commer

cial wet

feed

☐ raw meat

☐ table

leftovers

☐ other:

29. Is your dog fed in the kitchen?

○ yes

○ no

30. Does your dog come into contact with dogs from another household?

- Never
- Monthly
- Weekly
- Daily

31. Does your dog come into contact with farm animals?

	Never	Monthly	Weekly	Daily
Cattle				
Horses				
Sheep				
Goats				
Poultry				
Rabbits (b				

red for meat)

Pigs	○	○	○	○

32. Did you go to the vet with your dog in the past 6 months because of illness (not for yearly vaccination, deworming or treatment for fleas)

○ yes

○ no

33. If you did go to the vet with your dog in the past 6 months because of illness, please specify the reason why you did so:

[ ]

34. Was your dog hospitalized in the past 6 months because of illness? (not in case of castration or spaying)

○ yes

○ no

35. If your dog was hospitalized in the past 6 months because of illness, please specify the reason why it was hospitalized:

[ ]

36. Did your dog receive antibiotics in the past 6 months?

○ yes

○

n
o

37. If your dog received antibiotics in the past 6 months, please specify the reason why it got antibiotics:

38. Does your dog have a condition that results in a weakened immune system? (diabetes, cancer, Leukemia, …)

y
e
s

n
o

In the next part of the questionnaire, questions will be asked regarding the contact between you and your dog. Keep in mind that there are no wrong answers.

Contact between dog and its owner

Contact between dog and its owner

39. contact between you and your dog	Never	Sometimes	Often
Do you pet your dog? 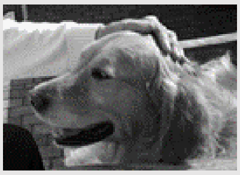			
Does your dog bite in your hand? (playful or out of aggression)			
Do you carry your dog?			
Does your dog push against your hand? 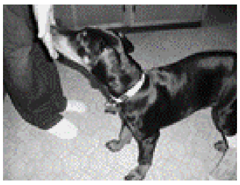			
Do you hug your dog?			
Does your dog come into your bed?			
Does the dog lie in your lap?			
Does the dog lick your hand?			
Does the dog lick your face?			

Does the dog smell your hand?	○		
Does the dog jump up against you?	○	○	○
Does the dog sit with you on the sofa?	○	○	○

40. contact between you and your dog	Never	Sometimes	Often
Does the dog eat out of your hand? (this also includes treats)	○	○	○
Does the dog eat out of your plate?	○	○	○
Does the dog drink out of the toilet?	○	○	○
Does the dog drink out of the sink?	○	○	○

40. contact between you and your dog			
Do you play fetch games with your dog?	○	○	○
Do you play tug-of-war games with your dog?	○	○	○
Does the dog sneeze in your face?	○	○	○
Does the dog sneeze on your hands?	○	○	○
Does the dog travel with you abroad?	○	○	○

42. Hygiene	Never	Sometimes	Often	N/A
Does your dog eat stool?	○	○	○	○
Does your dog defecate in the house?	○	○	○	○
Does your dog pee in the house?	○	○	○	○
Do you brush the teeth of your dog?	○	○	○	○
Do you clean the eyes of your dog?	○	○	○	○
Do you clean the ears of your dog?	○	○	○	○
Do you remove your dog's stool from the garden?	○	○	○	○
Do you wash your dog's blankets?	○	○	○	○
Do you wash your hands after you have touched your dog?	○	○	○	○
Do you wash your hands after you have removed the urine of stool from your dog?	○	○	○	○
Do you brush the dog?	○	○	○	○
Do you wash the dog in your bathroom	○	○	○	○

Contact between dog and its owner

Information Surveyor

39. Select the name of the surveyor

When the name is not in the drop-down menu, please write it down below:

Contact between dog and its owner

Thank you

Thank you for completing this survey. All information will be analyzed anonymously.

Supplementary Materials Part A: univariate logistic regression to the dog-owner contact score

To look for aspects influencing the dog-owner contact score, factors were created based on the information retrieved from the questionnaires. These factors represent general information of the respondent (dog owner) such as gender, age, place of residency but also include information regarding the owner's dog, such as age and size of the dog. After creating the factors, they were used in a univariate linear regression with the dog-owner contact score as dependent variable. Each factor was seen as an independent variable (binary, categorical, continuous) to look at the relation between this factor and the dependent variable. All tested factors are listed below in Table S1.

Table S1. The univariable regression model with the dog-owner contact score as dependent variable. Showing that gender, age and residency of the respondent as well as age and size of the dog are significantly associated with the dog-owner contact score. Statistical significance was assessed at $P < 0.2$. The table is based on 701 observations.

	Estimate	Standard Error	P-Value
**Gender Respondent**	−2.1	0.6	0.01
**Age Respondent**	−1.7	0.4	< 0.001
**Place of Residency**	−2.0	0.4	< 0.001
**Academic Degree**	−0.2	0.3	0.4
**Health**	0.06	1.2	1.0
**Working in Hospital**	−0.8	1.7	0.6
**Professional Contact with Animals**	−0.3	1.5	0.9
**Educational Contact with Animals**	0.4	1.0	0.7
**Age Dog**	−2.6	0.7	< 0.001
**Size Dog**	−3.2	0.4	< 0.001

© 2020 by the authors. Submitted for possible open access publication under the terms and conditions of the Creative Commons Attribution (CC BY) license (http://creativecommons.org/licenses/by/4.0/).
